# 

**Published:** 1879-10

**Authors:** 


No. 310. OCTOBER, 1879. PRICE 15 CTS.

TWENTY-SIXTH YEAR.

### HALL'S

### Journal of Health.

E. H. GIBBS, A.M., M.D., EDITOR,

NO. 141 EIGHTH STREET (NEAR BROADWAY) NEW YORK.

Terms, $1.50 a Year, or $1.00 for Eight Months. Postage paid.

Entered as Second-Class matter in the Post-office in New York.

